# Constraints to counting bioluminescence producing cells by a commonly used transgene promoter and its implications for experimental design

**DOI:** 10.1038/s41598-019-46916-z

**Published:** 2019-08-05

**Authors:** E. O. Mosaad, K. Futrega, I. Seim, B. Gloss, K. F. Chambers, J. A. Clements, M. R. Doran

**Affiliations:** 1 School of Biomedical Sciences, Institute of Health and Biomedical Innovation, Queensland University of Technology, Brisbane, Australia; 2 Australian Prostate Cancer Research Centre-Queensland (APCRC-Q), Translational Research Institute, Brisbane, Australia; 3 Biochemistry Division, Chemistry Department, Faculty of Science, Damietta University, Damietta, Egypt; 4 Comparative and Endocrine Biology Laboratory, Translational Research Institute-Institute of Health and Biomedical Innovation, School of Biomedical Sciences, Queensland University of Technology, Brisbane, Australia; 5 Integrative Biology Laboratory, College of Life Sciences, Nanjing Normal University, Nanjing, China; 6 Garvan Institute of Medical Research, Sydney, Australia; 7 Quadram Institute Bioscience, Norwich Research Park, Norwich, UK; 8 Mater Medical Research – University of Queensland, Translational Research Institute (TRI), Brisbane, Australia; 9 Australian National Centre for the Public Awareness of Science, Australian National University, Canberra, Australia

**Keywords:** Cellular imaging, Gene expression, Cancer microenvironment, Cancer models

## Abstract

It is routine to genetically modify cells to express fluorescent or bioluminescent reporter proteins to enable tracking or quantification of cells *in vitro* and *in vivo*. Herein, we characterized the stability of luciferase reporter systems in C4-2B prostate cancer cells in mono-culture and in co-culture with bone marrow-derived mesenchymal stem/stromal cells (BMSC). An assumption made when employing the luciferase reporter is that the luciferase expressing cell number and bioluminescence signal are linearly proportional. We observed instances where luciferase expression was significantly upregulated in C4-2B cell populations when co-cultured with BMSC, resulting in a significant disconnect between bioluminescence signal and cell number. We subsequently characterized luciferase reporter stability in a second C4-2B reporter cell line, and six other cancer cell lines. All but the single C4-2B reporter cell population had stable luciferase reporter expression in mono-culture and BMSC co-culture. Whole-genome sequencing revealed that relative number of luciferase gene insertions per genome in the unstable C4-2B reporter cell population was lesser than stable C4-2B, PC3 and MD-MBA-231 luciferase reporter cell lines. We reasoned that the low luciferase gene copy number and genome insertion locations likely contributed to the reporter gene expression being exquisitely sensitive BMSC paracrine signals. In this study, we show that it is possible to generate a range of stable and reliable luciferase reporter prostate- and breast- cancer cell populations but advise not to assume stability across different culture conditions. Reporter stability should be validated, on a case-by-case basis, for each cell line and culture condition.

## Introduction

Quantifying the number of a specific type in complex co-cultures, or in animals, requires that cells of interest are distinguishable from neighboring cells. To this end, it is increasingly common to genetically label cells such that they express a specific fluorescent or bioluminescent (e.g. luciferase) reporter proteins, thereby enabling their tracking and quantification. Luciferase-expressing cancer cells have been used previously to estimate their number in complex co-cultures^1–3^. For example, in *Nature Medicine*, McMillin and colleagues retrovirally transduced human multiple myeloma cells to express luciferase and used this as a reporter to quantify myeloma cells in co-culture with bone marrow-derived mesenchymal stromal cells (BMSC)^4^. It is increasingly recognized that non-cancerous cells in the tumor microenvironment play a significant role in tumor establishment, growth and drug response^5–8^. For these reasons, co-cultures are increasingly being utilize, and reporter-based co-culture assays use will increase in frequency.

The underlying assumption when using bioluminescence to estimate the number of cells in a co-culture, or in an animal, is that there is a linear relationship between the bioluminescence signal and the number of viable cells expressing the reporter protein (typically luciferase). This requires that expression and production of the luciferase enzyme is stable in cell populations over time, and across different culture conditions. This is analogous to the properties desired by a housekeeping gene in RT-qPCR^9^. It is common practice for research groups to calibrate their bioluminescence assays using a titration of luciferase-expressing cells in a mono-culture. The mono-culture titration data is next used to estimate the number of luciferase-expressing cells in a co-culture based on relative bioluminescence^10^.

The luciferase reporter construct, which is inserted into the genome of the labelled cell population in stable cell lines, is usually composed of at least two regions. The first region is a promoter designed to constitutively drive the expression of the reporter(s). The second region is the reporter, usually the gene sequence for bioluminescent luciferase, a fluorescent protein (for example red fluorescent protein, RFP; or green fluorescent protein, GFP), or both. The gene expression of the reporter is dependent on that of the promoter, and ideally the promoter activity should be uniform across all culture conditions^11^.

In our own work, we observed instances where the luciferase bioluminescence signal generated by C4-2B cells, an LNCaP-derived cell line isolated from metastatic prostate cancer cells from the lumbar spine of castrated mice^12,13^, was significantly greater when these cells were co-cultured with BMSC, compared to mono-culture. These observations suggested that a luciferase reporter may not be a viable method to estimate relative cancer cell number under co-culture conditions. Herein, we sought to better understand the utility and reliability of using the bioluminescence signal from luciferase transduced cells to estimate C4-2B cell numbers in BMSC co-culture. We compared bioluminescence signal, luciferase gene expression, and DNA content in C4-2B populations where the luciferase reporter appeared to be stable and in populations where the luciferase reporter appeared to be unstable. Next, we performed whole-genome sequencing to determine the genetic difference between the stable and unstable cell lines. Then, we characterized the stability of six additional luciferase reporter cell populations, driven by distinct gene promoters, in mono-cultures and BMSC co-cultures. Finally, we performed whole-genome sequencing on two additional stable reporter cell lines in an effort to determine if reporter stability could be predicted from sequence data.

## Materials and Methods

### Bone marrow-derived mesenchymal stromal cell isolation and characterization

Human bone marrow aspirates were collected at the Mater Hospital (Brisbane, Australia) from fully informed and consenting healthy volunteer donors. Ethical approval was granted through the Mater Health Services Human Research Ethics Committee and the Queensland University of Technology Ethics Committee (number 1000000938); in accordance with the Australian National Health and Medical Research Council’s Statement on Ethical Conduct in Research Involving Humans. Mononuclear cell isolation was achieved by density gradient centrifugation, using Ficoll-Paque Plus (GE Healthcare), as previously described^14^. Cells were maintained in low glucose Dulbecco’s modified Eagle’s medium (DMEM-LG; Gibco, Invitrogen) supplemented with 10% fetal bovine serum (FBS), 100 U/mL penicillin, and 100 μg/mL streptomycin (1% PS; Gibco) in a humidified incubator containing 5% CO_2_ with 2% O_2_ atmosphere at 37 °C. The isolated cells were characterized by flow cytometry for their expression of BMSC specific surface antigen panel (Supplementary Table 1). Mesodermal trilineage differentiation capacity was confirmed using the corresponding induction media for osteogenic, adipogenic, and chondrogenic differentiation; using methods described previously^15,16^ (Supplementary Fig. 1).

### Cancer cell line culture

Two prostate cancer cell lines were used. PC3 were obtained from the American Type Culture Collection (American Type Culture Collection, ATCC) and C4-2B were derived and generously shared by Dr. Chung^12,13^. Breast cancer cell lines (MDA-MB-231 and MCF-7) were kindly provided by Dr. Eloïse Dray and Prof Lisa Chopin (Queensland University of Technology). Cell lines were authenticated at the Genomic Research Centre (GRC; Brisbane, Australia) using Short Tandem Repeat (STR) analysis. Briefly, STR profiles were compared to the ATCC STR Database to verify cell line identity. All cultures were performed in DMEM-LG (Gibco, Invitrogen) supplemented with 10% FBS and 1% PS in a humidified incubator containing 5% CO_2_ with a 20% O_2_ atmosphere at 37 °C.

### Production of luciferase-tagged prostate cancer cell lines

All transduced cell lines are listed in Table 1, along with the terms used throughout this study to describe them. Luciferase expressing C4-2B cells (termed C4-2B-CMV1 in Table 1) were generated using the 3^rd^ generation ViraPower lentiviral gene expression system (Invitrogen), as described previously^17^. This cell line was a generous gift from Dr. Patrick Ling (previously employed at Queensland University of Technology). As our team did not produce this specific cell line, we did not have the original vector map. We performed PCR to validate that in the C4-2B-CMV1 genome (1) the luciferase gene was present, (2) there the CMV promoter was present, and (3) that the CMV promoter sequence flanked the luciferase gene. This was achieved using the following steps: DNA was collected from C4-2B-CMV1 cells using TRIzol reagent (Invitrogen) as per the manufacturers’ instructions. Extracted DNA was used in PCR using Platinum Taq DNA polymerase (Invitrogen). Suitable PCR primers for *GAPDH*, *Luc* genes, and the CMV promoter were designed and their sequences listed in Table 2. To validate that the CMV promoter flanked the luciferase gene, we used the CMV forward primer and the luciferase reverse primer to amply a region that overlapped with both the promoter and luciferase gene. The thermocycling conditions were as follows: a single 2 minutes initial denaturation at 95 °C; 35 cycles of 30 seconds denaturation at 95 °C, 30 seconds annealing at 55 °C, and 45 seconds extension at 72 °C, and a one-minute final extension at 72 °C. PCR products were visualized using a 2% ethidium bromide-agarose gel in Tris-borate-EDTA buffer (TBE buffer, pH 8.3), Gel loading dye, Purple (New England Biolabs), and HyperLadder 50 bp (Bioline). The agarose gel was visualized under ultraviolet light.

**Table 1:** List of cancer cell lines used with respective transduced promoters.

Parent cell line	Promoter-reporter	Referred to as	Construct source
C4-2B	CMV-Luc	C4-2B-CMV1	Plasmid (Dr. Patrick Ling^17^)
C4-2B	CMV-Luc-RFP	C4-2B-CMV2	Plasmid (Supplementary Fig. 3b)
C4-2B	MSCV-Luc-GFP	C4-2B-MSCV	Plasmid (Supplementary Fig. 3a)
C4-2B	EF1a-luc-GFP	C4-2B-EF1a	Plasmid (Supplementary Fig. 3c)
PC3	CMV-Luc-RFP	PC3-CMV2	Plasmid (Supplementary Fig. 3b)
MCF-7	EF1a-Luc-GFP	MCF-7-EF1a	Viral particles (AMSBIO, LVP020)
MDA-MB-231	CMV-Luc-RFP	MDA-CMV2	Plasmid (Supplementary Fig. 3b)
MDA-MB-231	MSCV-Luc-GFP	MDA-MSCV	Plasmid (Supplementary Fig. 3a)

**Table 2:** Primers and annealing temperatures used for standard PCR.

Gene	Sequence (5′-3′)	Annealing temperature (°C)	Primer concentration (nM)	Amplicon size (bp)
*GAPDH*	F GGGAGGTAGAGGGGTGATGT R TTCAGCTCAGGGATGACCTT	60.0	400	204
*firefly luciferase (Luc)*	F TGAAGAGATACGCCCTGGTT R CCAACACCGGCATAAAGAAT	59.8	400	198
*CMV*	F GCGTGGATAGCGGTTTGACT R CAATGGGGCGGAGTTGTTAC	60.1	400	124

The firefly luciferase (Luc) and CMV primer pairs were used to demonstrate that C4-2B-CMV1 cell line genome had both genes, and that these gene sequences were adjacent to each other in the genome. The CMV forward primer and the luciferase reverse primer were used to amply the adjacent sequence spanning from the CMV promoter into the luciferase gene. GAPDH was used as a positive control in the PCR reactions.

We subsequently generated a number of additional luciferase-expressing prostate cancer and breast cancer cell lines. MCF-7 breast cancer cells were transduced with commercial, pre-made 3^rd^ generation lentiviral expression particles (AMSBIO, LVP020) as per the manufacturers’ instructions. In these cells, luciferase and GFP were driven by the elongation factor 1 alpha (EF1a) promoter. Cultures were enriched for transduced cells by FACSorting (MoFlo Astrios; Beckman Coulter) for GFP^+^ cells. Cultures were validated to be stably GFP^+^ at subsequent culture time points via flow cytometer analysis.

Using Lentiviral particles manufactured in-house, we transduced C4-2B, MDA-MB-231, and PC3 with plasmids carrying luciferase and a fluorescent reporter genes. These lentiviral particles contained constructs designed to express luciferase and red fluorescent protein (Luc-RFP) or luciferase and green fluorescent protein (Luc-GFP). Constructs were purchased from Bioluminescence Imaging Vectors (BLIV, System Biosciences). The promoter and color combinations were cytomegalovirus (CMV) as CMV-Luc-RFP, murine stem cell virus (MSCV) as MSCV-Luc-GFP, or (EF1a) as EF1a-Luc-GFP. Please see Supplementary Fig. 3 for construct details. Viral particles were manufactured, and cells transduced, as described below.

Plasmids were produced using Stbl3 Chemically Competent *E.coli* (Invitrogen), as per the manufacturer’s instructions. Plasmids were purified using a NucleoBond Xtra EF plasmid purification kit (Midi EF, Macherey-Nagel). They were packaged in Lipofectamine 2000 (Invitrogen), and transfected into 293FT cells (Invitrogen) to produce viral particles. Cancer cells were next exposed to the viral particles in the presence of 8 µg/mL polybrene to facilitate transduction. Transduced cells were FACs sorted to enrich for GFP^+^ or RFP^+^ cells, yielding cell lines stably expressing Luc-RFP or Luc-GFP with one of three regulatory promoters (MSCV, CMV, or EF1a).

### DNA quantitation

The Quant-iT PicoGreen dsDNA assay (Invitrogen) was performed, as per the manufacturer’s instructions, to determine the quantity of double stranded DNA (i.e. genomic DNA) in each culture condition.

### Cell viability measurement

The AlamarBlue assay (Invitrogen) was used to measure the metabolic activity of cells. AlamarBlue reagent was added to the culture media at a final concentration of 3%. The plates were incubated for 1 hour at 37 °C, to permit reduction of the AlamarBlue reagent, and fluorescence read at 544 nm excitation and 590 nm emission (BMG Omega plate reader (BMG LABTECH)).

### Bioluminescence assay

For luciferase assays, D-luciferin (Promega) was added to the culture medium at a final concentration of 15 μg/mL, incubated at 37 °C for 15 minutes, and bioluminescence measured using a PHERAstar FS plate reader (BMG LABTECH). Data is presented as relative bioluminescence (RLU) compared to the control, unless stated otherwise. Direct comparison of the relative bioluminescence of C4-2B-CMV1 and C4-2B-CMV2 was performed by contrasting the signal generated by titrations of the two cell types. For these studies, cells were seeded in 96 well plates (4 replicates each) at densities of 500, 1000, 2000, 5000, 10,000, 20,000, and 50,000 cells/well. Cells were permitted to attach to the tissue culture plastic surface for 4 hours, and then D-luciferin was added to the culture medium at a final concentration of 15 μg/mL, cultures incubated at 37 °C for 15 minutes, and bioluminescence measured.

### Luciferase antibody staining

To validate the relative percentage of C4-2B-CMV1 and C4-2B-CMV2 cells that were expressing detectable levels of luciferase protein, cells were fixed, permeabilized, stained with anti-Luciferase, and characterized by flow cytometry. Cells were prepared using FIX & PERM Cell Permeabilization Kit (ThermoFisher). Cells were stained with anti-firefly Luciferase (Alexa Fluor 488, Abcam ab214950) as per the manufacturer’s instructions, then characterized on a LSRFortessa X-20 flow cytometer (BD Biosciences), and data analyzed using FlowJo software (TreeStar).

### Co-culture system

For direct co-cultures, BMSC (1 × 10^4^) were seeded in 96-well plates for 24 hours to permit adherence to the tissue culture plate. The following day, a titration of cancer cells was seeded either on the top of adherent BMSC (co-cultures) or into empty wells (control mono-cultures). For Transwell assays, BMSC (1 × 10^4^) were seeded into the top Transwell insert (Millicell culture inserts, Merck Millipore) and 9 × 10^4^ cancer cells seeded in the bottom wells of 24-well plates. Transwell insert pore sizes of 0.4 µm were employed to prevent the passing of BMSC through the Transwell membrane, and to enable independent quantification of the cell number on the top and bottom of the cultures at endpoint. Co-cultures were incubated for 0, 5, 24 hours, bioluminescence measured, and cells harvested. A parallel mono-culture was maintained as a control for every time point.

### Quantitative real-time RT-PCR (qRT-PCR)

To assess the stability of luciferase gene expression in mono-cultures and co-cultures, RNA was extracted from cancer cells grown in mono-culture and indirect co-culture using an RNAeasy Mini Kit (QIAGEN). Luciferase gene primer pairs were designed using Primer3Plus^18^ and were checked for specificity by querying the firefly (*Photinus pyralis*) luciferase gene (GenBank: M15077.1) and *Homo sapiens* genome using NCBI Primer-BLAST^19^. To optimize the housekeeping genes and luciferase gene primers for qRT-PCR, four primer concentrations were used with three different cDNA template amounts. The optimum primer concentrations were selected based on conditions yielding the greatest amplification efficiency.

All RNA samples were treated with *DNase I* (1 U/µL final concentration) in solution, at 37 °C for 30 minutes followed by 10 minutes incubation at 65 °C to deactivate the enzyme. Next, cDNA was generated from 500 ng total RNA using the SuperScript III First-strand synthesis kit (Invitrogen). We measured relative gene expression using Power SYBR Green PCR master mix (Applied Biosystems) on Viia7 Real Time PCR system (Applied Biosystems) (5 µL reactions on a 384-well plate; three technical replicates). Each condition had four biological replicates. Amplification was performed with an initial cycle of 50 °C for 2 minutes and 60 °C for 1 minute, 40 quantification cycles (with one cycle consisting of 95 °C for 15 seconds and 60 °C for 1 minute), followed by the thermal dissociation protocol for SYBR Green detection. Relative luciferase gene expression was normalized to the housekeeping gene ribosomal protein lateral stalk subunit P0 (*RPLP0*). The primers used are listed in Table 3 along with the annealing temperature and the primers’ final concentrations in each reaction.

**Table 3:** Primers and annealing temperatures used for qRT-PCR.

Gene	Sequence (5′-3′)	Annealing temperature (°C)	Primer concentration (nM)	Amplicon size (bp)
*RPLP0*	F TGTGGGCTCCAAGCAGATGCA R GCAGCAGTTTCTCCAGAGCTGGG	60.0	200	137
*firefly luciferase*	F GTGTTGGGCGCGTTATTTAT R TACGGTAGGCTGCGAAATGT	60.7	200	102

### Whole-genome sequencing

Genomic DNA sequencing was performed at the Garvan Institute’s Kinghorn Centre for Clinical Genomics (KCCG; Sydney, Australia). DNA was extracted from C4-2B-CMV1, C4-2B-CMV2, PC3-CMV2, and MDA-MSCV cell populations using TRIzol reagent (Invitrogen). Whole-genome sequencing was performed on the HiSeqX Ten sequencing platform (TruSeq Nano) using HiSeq X Reagent Kit v2.5. Transgene insertion mapping was performed using STAR^20^, and chimeric reads with at least 20 base pair overhang between luciferase (GenBank: M15077.1) and human reference genome (hg38) retained. Discordant read-pair mappings from BWA-MEM^21^ were used to independently verify the location of the transgene insertions. Insert locations were visualized using Circos^22^. Full sequence data is freely available upon request; please contact the authors.

### Statistical analysis

Results are displayed as the mean values of three independent experiments, each with four technical replicates, unless mentioned otherwise. Error bars represent standard deviation. Statistical significance of data was evaluated using two-way analysis of variance (ANOVA) in Prism v6.0 (GraphPad Software). *P*-values obtained in each comparison are represented by asterisks in graphs, as detailed in figure captions.

## Results

### Validation of C4-2B-CMV1 luciferase and CVM promoter proximity

The C4-2B-CMV1 was described in a previous publication^17^ and gifted to our laboratory. To validate that the CMV promoter was driving luciferase gene expression in the C4-2B-CMV1, we used PCR to demonstrate that the CMV and luciferase genes were in the genome (Supplementary Fig. 2a) and adjacent to each other (Supplementary Fig. 2b). In the PCR reaction, we used a forward primer specific to the CMV sequence and a reverse primer specific to the luciferase gene sequence. This strategy enabled the amplification of a fragment of approximately 600 bp that spanned from the CMV promoter into the luciferase gene sequence.

### C4-2B-CMV1 characterization in mono-culture and co-culture

**Figure 1:**
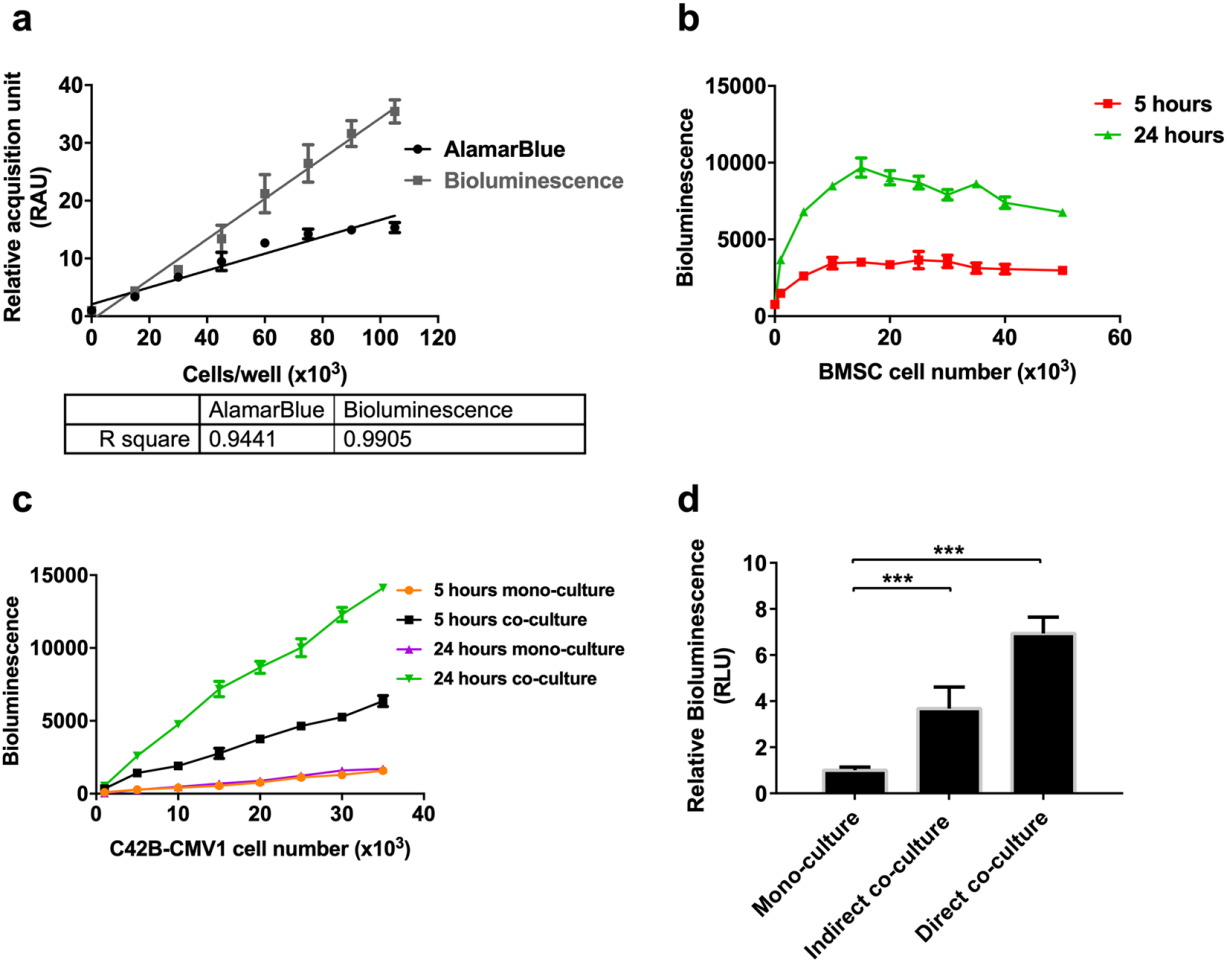
C4-2B-CMV1 behavior in mono-cultures and co-cultures with bone marrow-derived mesenchymal stromal cells (BMSC). (**a**) Comparison of luciferase and AlamarBlue assay readouts in C4-2B-CMV1 mono-cultures. **(b)** Ten thousand C4-2B-CMV1 cells were directly cultured with increasing cell number of bone marrow-derived mesenchymal stromal cells (BMSC). Bioluminescence was measured at the indicated 2 time points. Data was normalized to the values of the lowest BMSC cell density at the corresponding time point (*n* = 4). **(c)** Increasing numbers of C4-2B-CMV1 cells were cultures alone (mono-culture) or directly cultured with 10 × 10^3^ BMSC (co-culture) in 96-well plates. The graph represents the mean bioluminescence values of 2 independent experiments each having 3 replicate cultures (*n* = 2). **(d)** The relative bioluminescence values elevated after 5 hours of direct and indirect co-cultures of C4-2B-CMV1 and BMSC compared to mono-cultures. Three independent experiments each had four technical replicate cultures (*n* = 4) were performed (**A**–**D**). Statistical significance was performed using Student’s *t*-test (***P < 0.001).

BMSC were used in co-cultures. These cells demonstrated expected BMSC characteristics, including accepted CD marker profiles and tri-lineage differentiation capacity (see Supplementary Table 1 and Supplementary Fig. 1)^23^. We characterized the bioluminescence signal and AlamarBlue conversion of cultures established from different numbers of C4-2B-CMV1 cells (Fig. 1a). Both bioluminescence signal and AlamarBlue signal were linearly proportional to the number of C4-2B-CMV1 cells in mono-culture. When C4-2B-CMV1 cells were co-cultured with varying numbers of BMSC for 5 or 24 hours, the bioluminescence signal increased substantially in cultures containing BMSC (Fig. 1b). The bioluminescence signal increased with greater BMSC co-culture number up to 10 × 10^3^ BMSC, after which the bioluminescence signal stabilized. When titrations of C4-2B-CMV1 cell numbers were made with fixed numbers of BMSC in co-culture, a bioluminescence signal proportional to C4-2B-CMV1 cell number was evident (Fig. 1c). However, the linear relationship between bioluminescence signal and C4-2B-CMV1 cell number was significantly different for mono- and co-cultures at 5 and 24 hours. The slope of the curve was always greater for co-cultures, relative to time-matched (5 or 24 hours) mono-culture controls. At 5 hours, the bioluminescence signal from C4-2B-CMV1 cells was significantly greater when these cells were maintained either in direct co-culture (seeded on top of BMSC) or indirect co-culture (with BMSC in a Transwell assay), relative to mono-culture controls (Fig. 1d). Direct and indirect co-culture resulted in ~7-fold or ~4-fold increase in bioluminescence signal after 5 hours of culture, respectively. As C4-2B cells double approximately once every 48 hours^24^, the magnitude of bioluminescence signal increase after 5 hours could not be accounted for by cell proliferation.

### C4-2B-CMV1 versus C4-2B-CMV2 culture characterization

**Figure 2:**
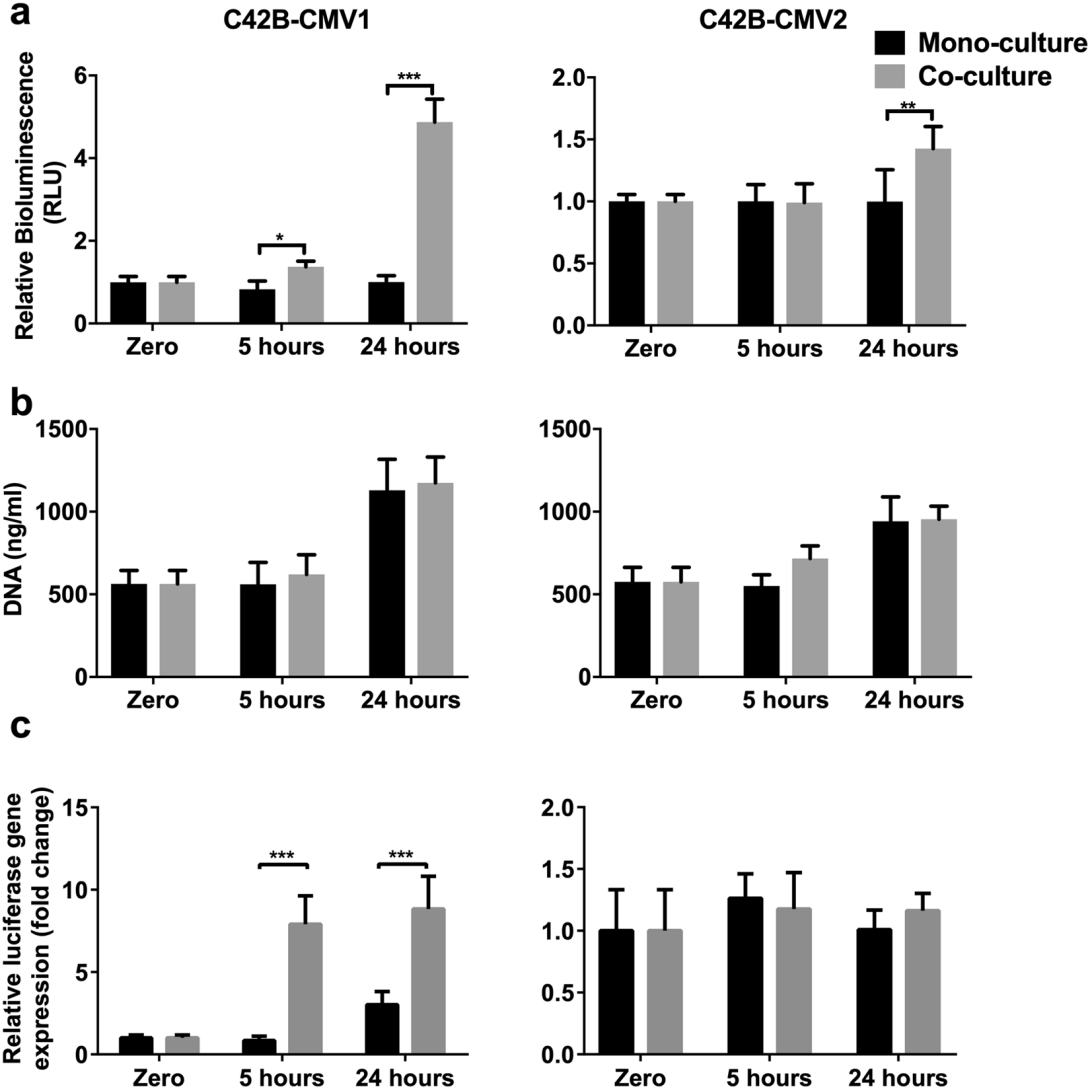
Luciferase gene expression is dependent on culture condition. C4-2B-CMV1 (left panel) and C4-2B-CMV2 cell lines (right panel) were either mono- or co-cultured with BMSC in a Transwell assay for the indicated time points. The relative bioluminescence (**a**), DNA content (**b**) and relative luciferase gene expression **(c)** were assessed. Three independent experiments each had four technical replicate cultures (*n* = 4) were performed. Statistical significance was determined using two-way ANOVA (*P < 0.05, **P < 0.001, ***P < 0.00001).

We compared the bioluminescence signal, DNA content, and luciferase gene expression in C4-2B-CMV1 versus C4-2B-CMV2 mono-cultures and co-cultures with BMSC in Transwell assay (Fig. 2). At 5 and 24 hours there was a significant increase in the bioluminescence signal from C4-2B-CMV1 cell co-cultured with BMSC (Fig. 2a). This was consistent with the data presented in Fig. 1. This increase correlated with a significant upregulation in luciferase gene expression (Fig. 2c). However, there was no corresponding increase in C4-2B-CMV1 cell culture DNA content in co-cultures, relative to mono-cultures. This suggested that the increase in luciferase gene expression and bioluminescence signal was not related to an increase in C4-2B-CMV1 cell number in co-cultures. Rather, luciferase gene expression and bioluminescence signal in C4-2B-CMV1 appeared to change independently of cell number in co-cultures.

A small, but consistent, increase in bioluminescence signal from C4-2B-CMV2 cells was detected after 24 hours of co-culture, relative to mono-culture controls. There was no measurable difference in luciferase gene expression or DNA content in C4-2B-CMV2 mono-cultures and BMSC co-cultures. C4-2B-CMV1 and C4-2B-CMV2 cell culture DNA content did increase over the 24-hour culture period, but the presence of BMSC did not measurably alter the rate of DNA content increase. Luciferase expression and bioluminescence signal was stable across mono- and co-culture conditions for C4-2B-CMV2 cells at 5 hours. By contrast, luciferase expression and bioluminescence signal were not stable across mono and co-culture conditions for C4-2B-CMV1 cells at 5 hours. We replicated these assays with different quantities of FBS or co-culture with parental C4-2B cells in place of BMSC (C4-2B cells not modified to express luciferase) to determine if a change in any soluble signaling environment could cause the artefact shown in Fig. 2. Neither changes in FBS concentration, nor co-culture with additional parental C4-2B cells, modified the bioluminescence signal (see Supplementary Fig. 4). These data indicated that factors specifically secreted by BMSC, not contained in FBS or secreted by C4-2B cells, caused the increase in bioluminescence signal from the C4-2B-CMV1 cells.

### C4-2B-CMV1 versus C4-2B-CMV2 relative bioluminescence and luciferase content

**Figure 3:**
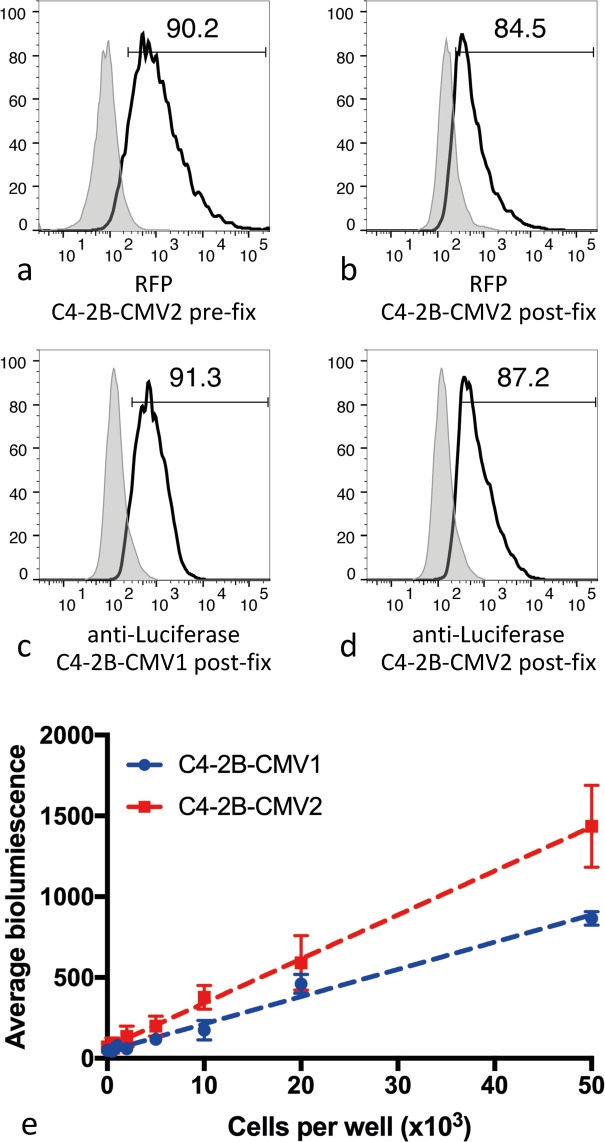
Detection of luciferase protein in discrete C4-2B-CMV1 and C4-2B-CMV2 cells, and comparison of population level relative bioluminescent signal. (**a**) RFP signal from C4-2B-CMV2 relative to wild-type C4-2B cells. (**b**) RFP signal from fixed and permeabilized C4-2B-CMV2 relative to wild-type C4-2B cells. (**c**) Anti-luciferase signal (Alexa Fluor 488) from fixed and permeabilized C4-2B-CMV1 cells, relative to wild-type C4-2B cell controls. (**d**) Anti-luciferase signal (Alexa Fluor 488) from fixed and permeabilized C4-2B-CMV2 cells, relative to wild-type C4-2B cell controls. (**e**) Relative bioluminescence signal intensity from titrations of C4-2B-CMV1 and C4-2B-CMV2 cells 4 hours after seeding (*n* = 4, error bars = 1 standard deviation). Regression analysis yielded a slope for the C4-2B-CMV1 cell population of 0.0168 ± 0.0085 and a slope of 0.0272 ± 0.0004 for the C4-2B-CMV2 cell population.

We reasoned that the observations in Fig. 2 could potentially result if only a small portion of the C4-2B-CMV1 cell population were stably transduced. To better understand this, and to determine the percentage of transduced cells, we quantified the proportion of C4-2B-CMV1 and C4-2B-CMV2 cells that contained the luciferase protein. This was performed using an antibody against luciferase, and characterization of the stained cells via flow cytometry. This was an important validation step, as the C4-2B-CMV1 cell line did not express a fluorescent reporter that would enable direct validation of cell transduction by tracking of the percent of fluorescent cells. Figure 3a–d show flow cytometry characterization of the C4-2B-CMV1 and C4-2B-CMV2 cell lines. Figure 3a shows direct flow cytometry analysis of C4-2B-CMV2 cells relative to wild-type C4-2B cells. In this analysis approximately 90% of C4-2B-CMV2 had an RFP signal greater than wild-type, suggesting that at least 90% of the C4-2B-CMV2 cells were stably transduced. Figure 3b shows that the RFP signal from the same cells decreased approximately 5% following treatment with the FIX & PERM Cell Permeabilization Kit (ThermoFisher). Fixation of cells is known to compromise RPF fluorescence^25^, and some signal loss following fixation was expected. Figure 3c,d show anti-luciferase (Alexa Fluor 488) signal from C4-2B-CMV1 and C4-2B-CMV2 cell lines, respectively, and relative to wild-type C4-2B cells also stained with anti-luciferase. Luciferase protein signal was present in the majority of both C4-2B-CMV1 and C4-2B-CMV2 cells. Because fixation is likely to influence antibody binding, we did not use this as a quantitative assay, but rather used it as a binary assay to indicate if individual cells in the bulk populations were likely to contain luciferase protein. We subsequently, used titrations of C4-2B-CMV1 and C4-2B-CMV2 cells to contrast the relative bioluminescence, and to infer the relative luciferase protein expressed by both cell populations. Figure 3e shows that the C4-2B-CMV2 cell population yielded a stronger bioluminescence signal than C4-2B-CMV1 cell population. Regression analysis demonstrated that the slope, or relative intensity per cell, for the C4-2B-CMV1 cell population was 0.0168 ± 0.0085 and 0.0272 ± 0.0004 for C4-2B-CMV2 cell population, equating to approximately 1.6-fold greater intensity from the C4-2B-CMV2 cell population. In summation, the majority of individual cells within the C4-2B-CMV1 cell population appeared to contain detectable levels of luciferase. By comparison, C4-2B-CMV1 cells were dimmer at the population level.

### C4-2B-CMV1 versus C4-2B-CMV2 genome characterization

**Figure 4:**
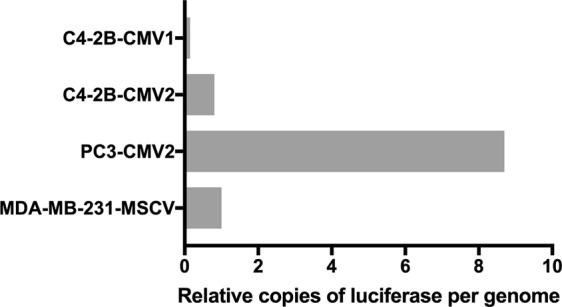
Using whole-genome sequencing of the reporter cell populations (at 30× coverage). We estimated the relative number of firefly luciferase gene insertions per genome. Chromosome numbers derived from previous karyotype analysis were to standardize insertion per genome estimates.

To gain insight into why the C4-2B-CMV1 and C4-2B-CMV2 cells behaved so differently, we conducted whole-genome sequencing of both populations (at 30× coverage). This process was also completed using two other cancer cell lines (PC3-CMV2 and MDA-MB-231-MSCV, described in Table 1) both shown to yield a stable bioluminescence signal in the presence of BMSC co-culture. All of the cancer cell lines (C4-2B, PC3, MDA-MB-231) sequenced in our study exhibit aneuploidy. Previous studies characterized C4-2B as having 83 chromosomes^24^, PC3 cells as having 62 chromosomes^26^, and MDA-MB-231 having a reported mean chromosome number between 65–69^27^. To account for the heterogeneity within the individual cell lines, and to standardize for the number of additional chromosomes in the different cell lines, we estimated the number of luciferase gene coding reads on a per genome basis. Assuming a standard human genome of 46 chromosomes, we plotted the relative number of chimeric read mapping between the human genome and firefly luciferase (Fig. 4). Chromosome numbers derived from previous karyotype analysis were to standardize insertion per genome estimates^24,26,27^. Once standardized, first we observed that the luciferase sequence was approximately 5-times more abundant in C4-2B-CMV2 cells relative to C4-2B-CMV1 cells on a per genome basis (Fig. 4, top two bars). When we contrasted C4-2B-CMV1 and C4-2B-CMV2 against PC3-CMV2 and MDA-MSCV reporter cell lines, we found that the luciferase sequence abundance was similar or greater in frequency to that observed in the C4-2B-CMV2 cell line on a per genome basis.

To better understand factors, in addition to the luciferase sequence abundance, that might impact on reporter stability we used chimeric read mapping between the human genome and firefly luciferase gene sequence to map sites with evidence of CMV-luciferase insertion into the C4-2B genomes. This analysis mapped 4 CMV-luciferase insertion sites in the C4-2B-CMV1 cell population, while it mapped 84 insertion sites in the C4-2B-CMV2 cell population (Supplementary Fig. 5). Cumulatively, the unstable luciferase reporter in C4-2B-CMV1 cells had a reduced number of insertions per genome and a reduced number of insertion sites relative to comparable stable C4-2B-CMV2 cells.

### Stability of luciferase reporters in other breast and prostate cancer cell lines

**Figure 5:**
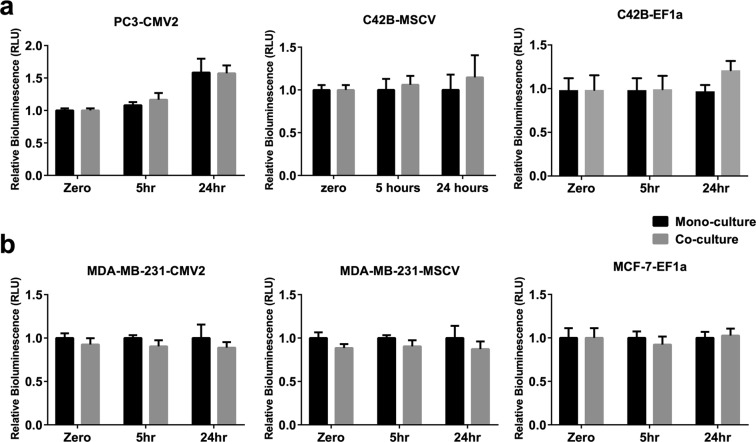
Luciferase reporter expression can be stable in prostate and breast cancer cells populations. (**a**) Prostate cancer cell lines (PC3 and C4-2B) and **(b)** breast cancer cell lines (MDA-MB-231 and MCF-7) cells were transduced by lentiviral particles to express luciferase driven by CMV2, MSCV or EF1a promoters. The bioluminescence was measured at the indicated time points of mono-cultures and co-cultures with bone marrow-derived mesenchymal stromal cells (BMSC) and normalized to the corresponding values at time point zero. Three independent experiments each had four technical replicate cultures (*n* = 3) were performed.

To gain a general understanding of the stability of the luciferase reporter in BMSC co-cultures with prostate and breast cancer cell lines, we generated and characterized the behavior of six additional reporter cell populations. We compared their relative bioluminescence signal in mono-cultures and in co-cultures with BMSC. We found that all six cell lines tested had stable luciferase reporter expression at both 5 and 24 hours (Fig. 5). The cell lines and the different promoters used to drive luciferase expression are included in the cell population name and described in the figure caption. Prostate cancer reporter cell lines were generated from parental PC3 and C4-2B cells. Breast cancer reporter cell lines were generated from parental MDA-MB-231 and MCF-7 cells. Luciferase gene expression was driven by CMV2, MSCV, or EF1a promoters.

PC3-CMV2 and MDA-MB-231-MSCV, for which relative luciferase gene insertion sites per genome are detailed in Fig. 4, were both found to be stable in mono-cultures and in co-cultures with BMSC. Their relative number of luciferase gene insertions per genome were found to be greater than the number of luciferase gene insertions in the unstable C4-2B-CMV1 cell population.

## Discussion

Luciferase reporter systems are increasingly used to estimate luciferase-expressing cancer cell number in co-cultures^4,8,28^ and in animal models^29,30^. This approach assumes that luciferase expression is stable under different culture conditions and within animal models. Most studies do not validate reporter stability in co-culture^4,8,28^. Our data (Fig. 5) suggest that in most cases, reporter cell lines produce a bioluminescence signal that is proportional to the reporter cell number in mono-culture and co-culture with BMSC. However, in some cases (Figs 1 and 2) the bioluminescence signal generated by luciferase reporter cells can be significantly different. We made this observation with a population of C4-2B cells transduced to express luciferase driven by the CMV promoter (we described this population as C4-2B-CMV1).

Here, we report on the stability of luciferase reporters in a number of prostate and breast cancer cell lines maintained in mono-culture or co-culture with BMSC. In mono-culture, both cell lines (C4-2B-CMV1 and control matched C4-2B-CMV2) generated strong bioluminescence signals that were proportional to the number of cells in culture (Fig. 3e). Additionally, the majority (>85%) C4-2B-CMV1 and C4-2B-CMV2 cells stained positively with anti-luciferase, suggesting that most cells contained detectable quantities of the firefly luciferase protein (Fig. 3c,d). Unlike monocultures, the bioluminescence signal from the C4-2B-CMV1 cell population increased 4 to 7-fold within 5 hours with indirect and direct co-culture with BMSC, respectively (Fig. 2). This increase in bioluminescence signal was not associated with an increase in cell number, indicating that the C4-2B-CMV1 reporter population cannot be used to estimate relative cell numbers in different culture conditions. This unexpected behavior might not be detected unless standard curves were generated simultaneously in mono- and co-culture conditions. This may explain why previous investigations in the literature have not reported this anomaly.

When the genomes of C4-2B-CMV1 and C4-2B-CMV2 were sequenced and compared we found that there were 4 versus 84 CMV-luciferase genome insertion sites, respectively (Supplementary Fig. 5) and a lower relative number of luciferase insertions (corrected for chromosome number; Fig. 4). The low number of CMV-luciferase genome insertion sites in the C4-2B-CMV1 cell population appears to render the cells more sensitive to the modified BMSC co-culture environment. We subsequently sequenced two additional stable luciferase reporter cell lines (PC3-CMV2 and MDA-MB-231-MSCV) and found that they also had a greater number of luciferase gene insertions per genome than the unstable C4-2B-CMV1 cell population (Fig. 4). It is worthwhile to consider that the generation of reporter cell lines involves selection for stably transduced cells. This selection process can be initiated from a small starting cell population, low initial transduction efficiency, and possibly iterative enrichment (i.e. sequential FACSorting of cultures to select for stably induced cell populations). Inefficiencies in each step could potentially select for a clone or clones with a small number of reporter gene sequences per genome. Additionally, cancer cell lines are known to be heterogenous^31,32^, and selection for stably transduced cells may yield a population that is not representative of the heterogenous parental population. Taken together, it is clear that a number of factors determines the success of a transduction process aimed at developing a cell line that stably expresses a reporter gene and is representative of the parental population.

Our data could be interpreted as indicating that a greater number of luciferase insertions per genome yields a reporter cell population more likely to be stable across a range of culture conditions. Indeed, it has been shown that the CMV promoter can be repressed or hyper-activated in various ways^33–35^. Thus, it is rational to assume that if the reporter construct is only located at a few sites within the genome it may be hypersensitive to small changes in the culture microenvironment. However, attempting to define a threshold of number of insertions that will yield a stable reporter cells may not be rational, as stability is likely a function of insert number and insert location(s) in the genome. Insert number and location(s) can be determined via sequencing of reporter cell lines, and mapping of insertion sites, but this remains costly (our cost was AUD $2,000 per genome, plus bioinformatics) and laborious.

We were not able to determine which signal(s) derived from the BMSC co-cultures modified luciferase expression in the unstable C4-2B-CMV1 cell population. BMSC are known to secrete a plethora of trophic factors that can influence cell behavior^36^, their secretions are thought to likely influence prostate cancer metastasis^37^, and it was in studying these interactions that we identified the phenomenon described in this paper. In an attempt to mimic the C4-2B-CMV1 cell population response to BMSC secretions, we tried unsuccessfully supplementing medium with growth factors (data not shown) or with additional FBS, which is known to contain many factors (See Supplementary Fig. 4). The broad range of signal molecules secreted by MSC was described as a “drug store” by Caplan^36^, and it may be that multiple factors in combination are required to generate the response observed in co-culture with BMSC.

Because knowing the number or the site of insertions will not definitively predict if a reporter is stable across multiple culture conditions, we recommend generating control cultures in each condition, and directly evaluating reporter stability in the manner we described above. Indeed, we also demonstrate that luciferase-expressing cell populations driven by a suitable promoter can function as excellent reporter systems in mono and co-cultures. In Fig. 5 we show examples of three different breast and prostate cancer cell lines that express luciferase driven by different promoters that yield a stable reliable and signal in both mono-cultures and BMSC co-cultures. While the probability of having experiments confounded by an unstable reporter is low, we warrant caution in interpreting results when the experimental conditions may directly impact the biology of the reporter cell population.

In summary, we show that a number of promoter-luciferase and cell combinations can be used to generate a reliable reporter cell line for use in mono- and co-cultures. We also show that, in some instances, reporter cell lines can be unreliable. Reliability is likely proportional to the number of reporter insertion sites per genome. However, as it is expensive to sequence each reporter cell line and count the number of insertion sites, direct sequencing of reporter cell genomes is likely not a preferred way to predict reporter stability. Furthermore, greater-than-a-threshold-number of reporter insertion sites might not reliably equate to reporter stability. Instead, we recommend comparing reporter stability across a range of culture conditions before proceeding with the intended study. Luciferase reporter cells are powerful tools, but stability across culture conditions should never be assumed.

## Supplementary information

Supplementary Data
