# Supplementary material for: Constraints to counting bioluminescence producing cells by a commonly used transgene promoter and its implications for experimental design
Source: Sci Rep. 2019 Aug 5;9:11334. doi: 10.1038/s41598-019-46916-z (PMC6683182; doi:10.1038/s41598-019-46916-z)
Supplement: Supplementary file 1 — Supplementary Data [file 41598_2019_46916_MOESM1_ESM.pdf]

Constraints to counting bioluminescence producing cells by a commonly used transgene promoter and its implications for experimental design

Mosaad, E. O.; Futrega, K.; Seim, I.; Gloss, B.; Chambers, K. F.; Clements, J. A.; Doran, M. R.*

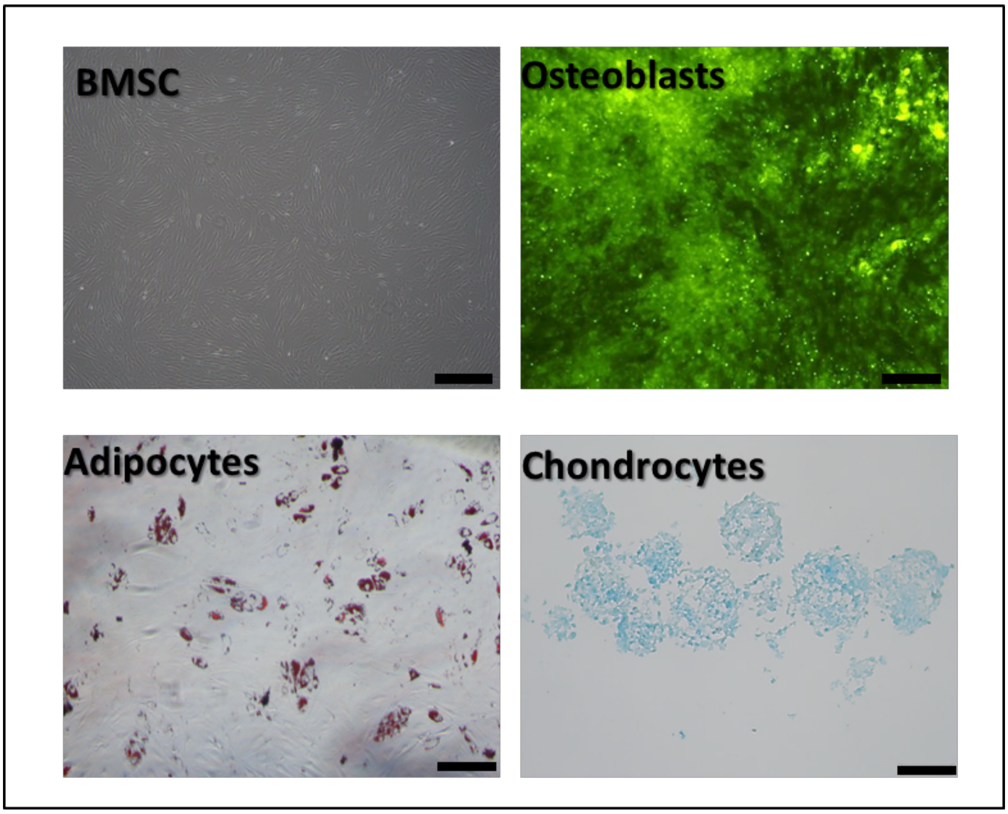

Supplementary Figure 1. Characterization of bone marrow-derived mesenchymal stromal cells (BMSC) trilineage differentiation. After isolation of BMSC, cells were seeded in monolayers for osteogenesis and adipogenesis induction; and in 3-dimensional cell aggregates for chondrogenesis induction. After 14 days, cells were fixed and stained with OsteoImage (green fluorescence staining of hydroxyapatite matrix, Osteoblasts), Oil Red O staining (red lipid vacuole staining, Adipocytes) or Alcian blue staining (blue staining of glycosaminoglycan matrix, Chondrocytes). Positive staining indicated mesodermal differentiation potential. Scale bars = 100 µm.

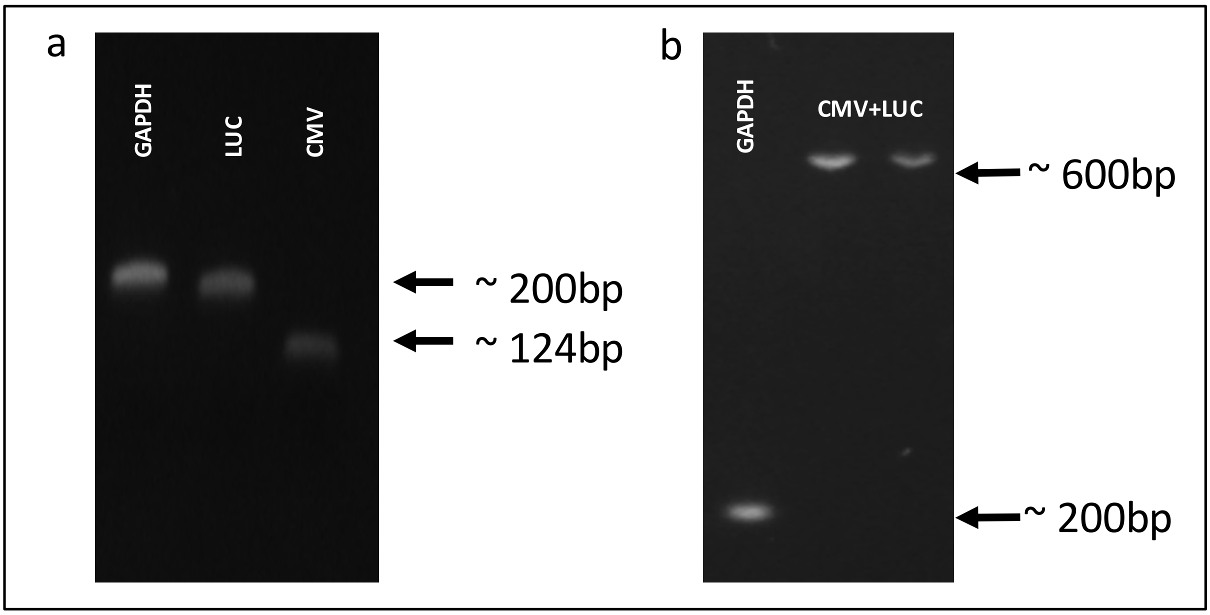

Supplementary Figure 2. PCR demonstrating a) the CMV promoter and luciferase gene are inserted in the C4-2B-CMV1 genome; and b) the CMV promoter and luciferase gene are adjacent in the C4-2B-CMV1 cell line. A band of approximately 600-base pair (bp) fragment which spans the two gene sequences. Control is the PCR reaction product from *GAPDH* (204 bp).

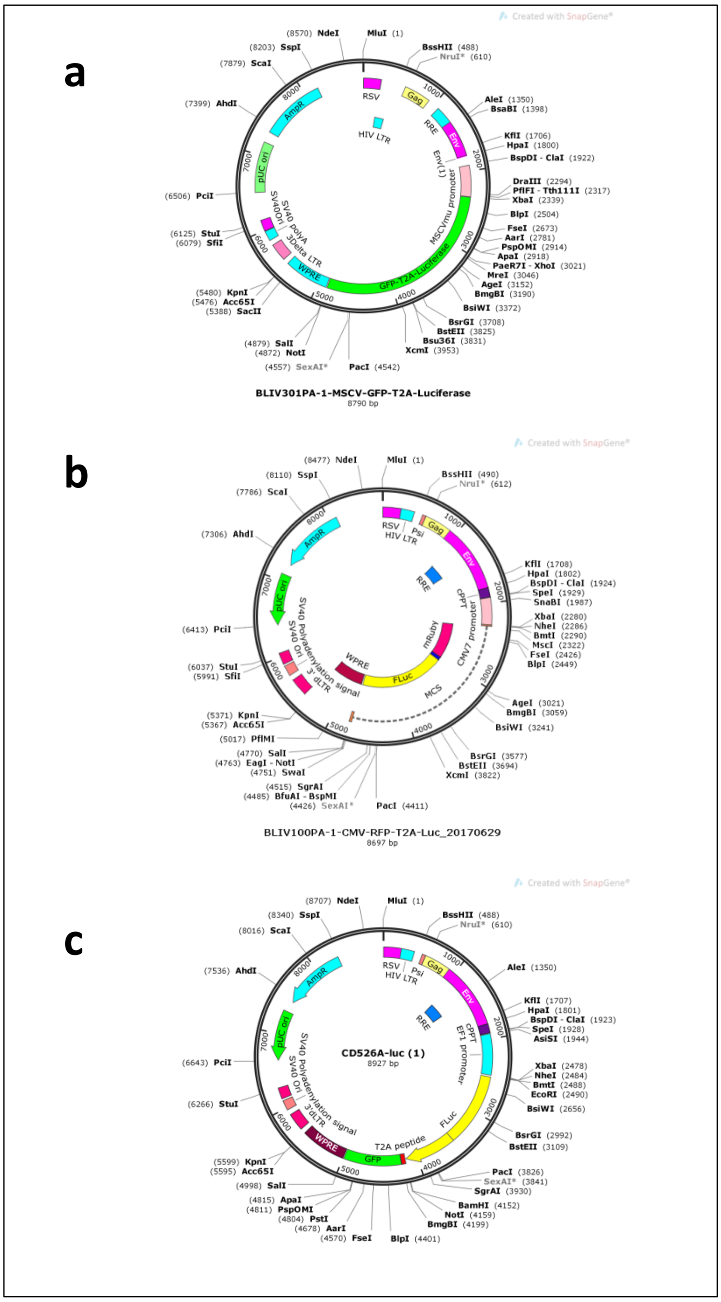

Supplementary Figure 3. Restriction maps of plasmids used in lentiviral particles production to transduce cells with luciferase gene with **(a)** MSCV, **(b)** CMV and **(c)** EF1a promoter regions. All plasmids were designed by System Biosciences (Bioluminescence Imaging Vectors, BLIV).

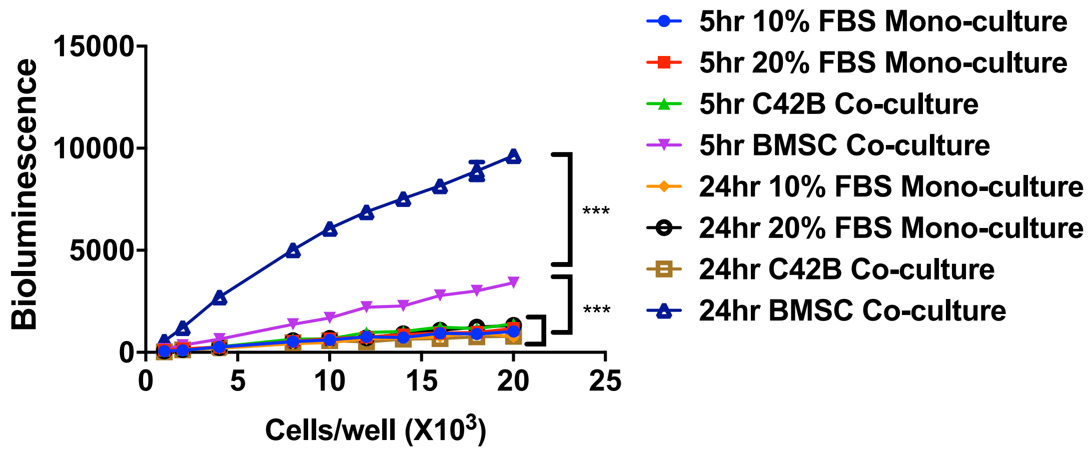

Supplementary Figure 4. The luciferase assay of C42B-CMV1 cells in mono- or co-culture with C42B-CMV1 cells or BMSC, or with different titrations of FBS. Increasing numbers of C42B-CMV1 cells were cultured in maintenance culture media (10% FBS Mono-culture), maintenance culture media supplemented with 20% FBS (20% FBS Mono-culture), directly cultured with 10×10^3 parental un-tagged C42B cells (C42B co-culture) or directly cultured with 10×10^3 BMSC (BMSC Co-culture) in 96-well plates. The bioluminescence was measured after 5 and 24 hours of culture establishment. Only co-culture with BMSC (open and closed purple triangles) resulted in an increase in bioluminescence signal. The graphed data represents the mean bioluminescence values of 6 replicate cultures at each time point and condition. Statistical significance was calculated using two-way ANOVA (*** $P < 0.001$).

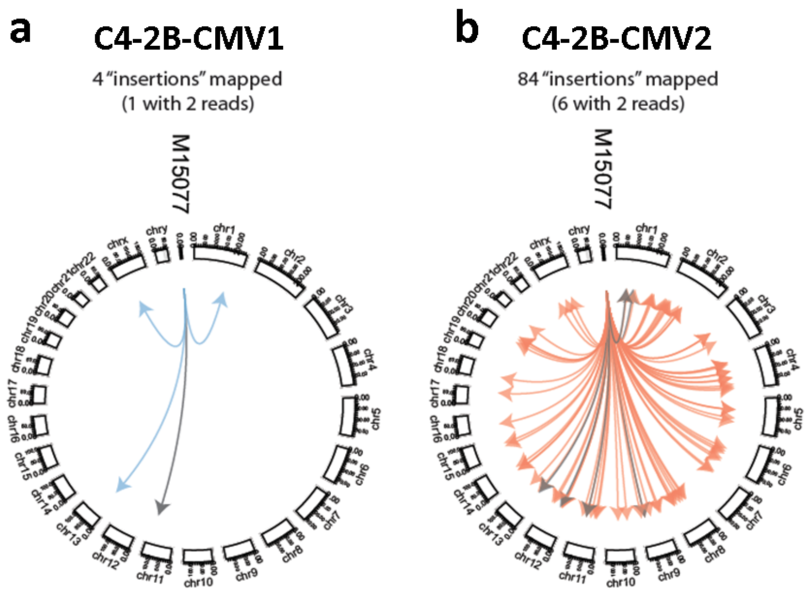

Supplementary Figure 5. Luciferase gene insertion map. Whole-genome sequencing data analysis of **(a)** C4-2B-CMV1 and **(b)** C4-2B-CMV2 revealed the number of insertion sites is 4 and 84 insertions, respectively.

Supplementary Table 1. List of bone marrow-derived mesenchymal stromal cell (BMSC) expression profile for cellular markers.

Marker	Reactivity*
CD45, CD34, CD271, HLA-DR	-ve
CD90, CD73, CD105, CD44, CD146	+ve

* (+ve) means > 95% and (-ve) means < 5% of the cell population.
